# Beneficial Effects of Testosterone Therapy on Functional Capacity, Cardiovascular Parameters, and Quality of Life in Patients with Congestive Heart Failure

**DOI:** 10.1155/2014/392432

**Published:** 2014-07-06

**Authors:** Ahmad Mirdamadi, Mohammad Garakyaraghi, Ali Pourmoghaddas, Alireza Bahmani, Hamideh Mahmoudi, Mojgan Gharipour

**Affiliations:** ^1^Islamic Azad University, Najafabad Branch, Isfahan, Iran; ^2^Heart Failure Research Center, Isfahan Cardiovascular Research Institute, Isfahan University of Medicine Science, Isfahan, Iran; ^3^Cardiac Rehabilitation Research Center, Isfahan Cardiovascular Research Institute, Isfahan University of Medicine Science, Isfahan, Iran; ^4^Isfahan Cardiovascular Research Center, Isfahan Cardiovascular Research Institute, Isfahan University of Medicine Science, Isfahan, Iran

## Abstract

*Background.* According to the present evidences suggesting association between low testosterone level and prediction of reduced exercise capacity as well as poor clinical outcome in patients with heart failure, we sought to determine if testosterone therapy improves clinical and cardiovascular conditions as well as quality of life status in patients with stable chronic heart failure. *Methods.* A total of 50 male patients who suffered from congestive heart failure were recruited in a double-blind, placebo-controlled trial and randomized to receive an intramuscular (gluteal) long-acting androgen injection (1 mL of testosterone enanthate 250 mg/mL) once every four weeks for 12 weeks or receive intramuscular injections of saline (1 mL of 0.9% wt/vol NaCl) with the same protocol. *Results.* The changes in body weight, hemodynamic parameters, and left ventricular dimensional echocardiographic indices were all comparable between the two groups. Regarding changes in diastolic functional state and using Tei index, this parameter was significantly improved. Unlike the group received placebo, those who received testosterone had a significant increasing trend in 6-walk mean distance (6MWD) parameter within the study period (*P* = 0.019). The discrepancy in the trends of changes in 6MWD between study groups remained significant after adjusting baseline variables (mean square = 243.262, *F* index = 4.402, and *P* = 0.045). *Conclusion.* Our study strengthens insights into the beneficial role of testosterone in improvement of functional capacity and quality of life in heart failure patients.

## 1. Introduction

Heart failure is a serious cardiovascular condition leading to life-threatening events, poor prognosis, and degradation of quality of life. In some studies, considerable morbidity and excessive mortality with a rate up to 30% has been annually exhibited [1]. In this regard, treatment and management of affected patients particularly those with progressive left ventricular dysfunction are a main problem and thus the use of single-drug treatment protocols or preventive approaches is mostly unpromising [2]. Recently, a noticeable evolution of therapeutic concepts has taken place with a variety of cardiac and hormonal drugs with the aim of improving patient's survival, preventing sudden death, and improving quality of life [3, 4]. In a significant proportion of heart failure patients, testosterone deficiency as an anabolic hormonal defect has been proven and identified even in both genders [5]. This metabolic and endocrinological abnormality is frequently associated with impaired exercise tolerance and reduced cardiac function [6]. For this reason, combination therapy with booster cardiovascular drugs and testosterone replacement therapy might be very beneficial in heart failure patients. The physiological pathways involved in these therapeutic processes have been recently examined. First, elevated level of testosterone following replacement therapy is major indicator for increase of peak VO_2_ in affected men with heart failure explaining improvement of exercise tolerance in these patients [7, 8]. Furthermore, testosterone replacement therapy can reduce circulating levels of inflammatory mediators including tumor necrosis factor *α* (TNF-*α*) and interleukin (IL)-1*β*, as well as total cholesterol in patients with established simultaneous coronary artery disease and testosterone deficiency [9, 10].

According to the present evidences suggesting association between low testosterone level and prediction of reduced exercise capacity as well as poor clinical outcome in patients with heart failure, we sought to determine if testosterone therapy improves clinical and cardiovascular conditions as well as quality of life status in patients with stable chronic heart failure.

## 2. Methods

This study was designed as a double-blind, placebo-controlled trial, with 50 male patients who suffered from congestive heart failure that is defined as an ejection fraction less than 40% on echocardiogram or clinical heart failure according to the Framingham's criteria for diagnosis of heart failure [11, 12] and recruited from 2011 to 2013. Inclusion criteria were age range 50 to 70 years and suffering congestive heart faire. Exclusion criteria were history or symptoms of adrenal or gonad disorders, recent history of unstable angina, myocardial infarction, uncontrolled hypertension, severe liver diseases, kidney disease, erythrocytosis (serum hematocrit > 50%), prostatic disorders, or international prostate symptom score (IPSS) more than 20. This research was approved by the Research and Ethics Committees at Isfahan University of Medical Sciences. Structured baseline interviews were carried out by a research assistant within hospitalization including questions about social demographic characteristics and as well as cardiovascular risk factors. Conventional cardiac risk factors were identified, including current smoking history (patients who regularly smoke tobacco product/products one or more times per day or have smoked in the 30 days prior to admission) [13], hypercholesterolemia (total cholesterol ≥ 5.0 mmoL/l, HDL-cholesterol < 1.0 mmoL/l in men or <1.1 mmoL/l in women, and triglycerides ≥ 2.0 mmoL/l) [14], family history of CAD (first-degree relatives before the age of 55 in men and 65 years in women) [15], hypertension (systolic blood pressure ≥ 140 mmHg and/or diastolic ≥ 90 mmHg and/or on antihypertensive treatment) [16], and diabetes mellitus (symptoms of diabetes plus at least one of the following: plasma glucose concentration ≥ 11.1 mmoL/l, fasting plasma glucose ≥ 7.0 mmoL/l, and 2-hpp ≥ 11.1 mmoL/l) [17]. Anthropometric parameters were measured using standard protocols. Functional limitation was assessed clinically according to the New York Heart Association (NYHA) classification and functional capacity was assessed by means of six-minute walk test (6MWT), that is, a symptom-limited exercise test involving a progressive increase in walking speed to maximal exercise tolerance [18]. Morphologic and functional changes in cardiac structure and function were assessed using an ultrasound device and dedicated 3.5 MHz phased-array ultrasonic transducer according to the American Society of Echocardiography guidelines for measuring left ventricular end systolic and end diastolic dimensions and volumes as well as systolic and diastolic functions, left ventricular filling pressure, and Tei index [19]. For assessment of quality of life state, the Short Form Health Survey (SF-36) questionnaire was used that the total score was calculated based on eight physical and psychological subscales and with a higher score indicating a better QOL [20]. Depressive symptoms were assessed using the Persian version of the Beck Depression Inventory (BDI) that was validated among Iranian cardiovascular population with the* Cronbach's alpha* of 0.92 for reliability and intraclass correlation coefficient of 0.72 for validity assessment [21]. BDI is a self-reported questionnaire, which is used for routine assessment of the severity of depression in psychiatric patients and for the screening for depressive symptoms in the general population. BDI consists of 21 items that cover emotional, behavioral, and somatic symptoms. The total score ranges from 0 to 63 points (scored 0–3 for each item) [22]. In this study, the 6 min walk test was conducted according to a standardized protocol and the 6 min walk distance (6MWD) was measured. Upper extremity strength was also assessed according to grip strength using the dynamometer. Grip strength involved two trials on each hand. The mean value of the two trials was obtained. For this analysis, the maximum strength in either hand was used for upper extremity strength [23]. Patients were randomized to receive an intramuscular (gluteal) long-acting androgen injection (1 mL of testosterone enanthate 250 mg/mL) once every 4 weeks for 12 weeks or receive intramuscular injections of saline (1 mL of 0.9% wt/vol NaCl) with the same protocol. Hence, all patients received 3 injections over the 12-week period. Measurements to the study including assessment of echocardiographic parameters, quality of life and depression states, and muscle strength were performed before intervention and also as endpoint after 12 weeks. Also, the changes in body weight, functional capacity, and hemodynamic indicators were assessed at baseline and also at the end of each month in both groups and compared. Results were reported as mean ± standard deviation (SD) for the quantitative variables and percentages for the categorical variables. The groups were compared using the Student's *t*-test or Mann-Whitney *U* test for the continuous variables and the chi-square test (or Fisher's exact test if required) for the categorical variables. The changes in the study parameters within study period were assessed and compared using the repeated measure ANOVA test. In this analysis, the effect size (indicated by *F*-index) indicated within- and between-subjects variability. The interpretation of the *F*-index is considered large (or significant) if it is higher than 0.40, medium if it ranged between 0.25 and 0.40, and small if it is lower than 0.25. For determining differences in the trends of study parameters between the two groups, the results of the repeated measure ANOVA test were adjusted for baseline variables. In this regard, *P* values of 0.05 or less for each variable indicated discrepancy in the time trend of the variable between the two groups. The difference in response to treatment protocols across the groups was assessed using a multivariable logistic regression model considering baseline data as probable independent confounders. The beta coefficient in this model was used to compare the strength of a predictor within the model to predict the dependant variable or outcome so that the higher beta indicates more power of the variable to predict outcome. Because beta coefficient is potentially affected by the standard error of the variable, the standardized beta adjusted for this error is considered. *P* values of 0.05 or less were considered statistically significant. All the statistical analyses were performed using SPSS version 19.0 (SPSS Inc., Chicago, IL, USA) and SAS version 9.1 for Windows (SAS Institute Inc., Cary, NC, USA).

## 3. Results

**Figure 1:**
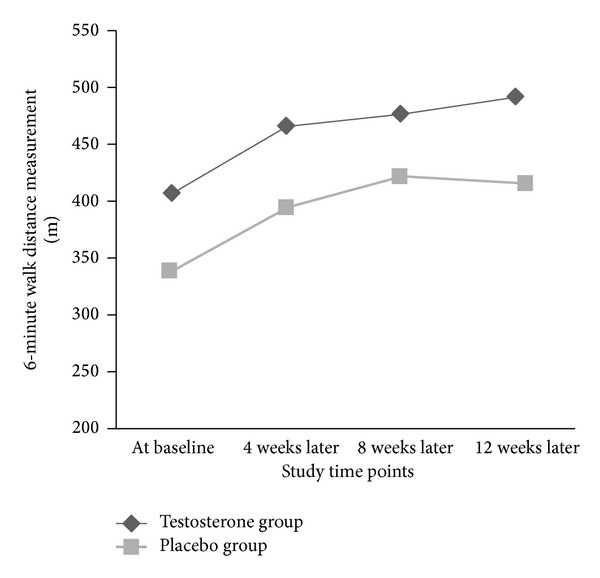
Trend of the changes in 6-minute walk distance (6MWD) parameter (in meter) in intervention and placebo groups.

Comparing baseline variables and clinical parameters across the two groups who received testosterone or placebo (Table 1) did not show any significant difference, except for 6MWD that was higher in the testosterone group. During the 12-week study period, no significant differences were revealed in the trend of the changes in hemodynamic parameters including systolic and diastolic blood pressures as well as heart rate between the two groups (Table 2). Also, the changes in body weight were comparable between the groups, while, unlike the group received placebo, those who received testosterone had a significant increasing trend in 6MWD parameter within the study period (6MWD at baseline was 407.44 ± 100.23 m and after 12 weeks of followup reached 491.65 ± 112.88 m following testosterone therapy, *P* = 0.019). According to post hoc analysis, the mean 6-walk distance parameter was improved at three time points of 4 weeks, 8 weeks, and 12 weeks after intervention compared with baseline; however no differences were found in this parameter at three postintervention time points. The discrepancy in the trends of changes in 6MWD between study groups remained significant after adjusting baseline variables (mean square = 243.262, *F*-index = 4.402, and *P* = 0.045) (Table 3 and Figure 1). The muscle strength was gradually increased in intervention group but not in control; however, this trend was not different across the two groups (Table 2). Among cardiovascular parameters assessed by echocardiography, no differences were observed between the patients who were prescribed testosterone and those who received placebo from baseline to end of the study time (Table 4). Also, regarding changes in diastolic functional state and using Tei index, this parameter was significantly improved. However, quality of life score was significantly improved in former group after adjustment for confounders using the multivariate linear regression modeling (Table 5).

## 4. Discussion

Hormonal metabolic impairment is a main part of fundamental pathophysiological features in heart failure patients. These endocrinological changes lead to serious clinical and prognostic consequences in these patients. On the top of these variations, abnormalities in the serum level of testosterone have been especially more paid attention to. Reducing testosterone level has been identified as the fundament of andropause or male menopause characterized by the slow but steady reduction of the production of the hormones testosterone and dehydroepiandrosterone in middle-aged men. This phenomenon seems to be more progressive in heart failure state leading inappropriate physical, cardiovascular, or psychological consequences. In a recent report, deficiencies in circulating testosterone were reported in 13% of heart failure patients that the reduced circulating testosterone was associated with lower heart rate variability and depleted baroreflex sensitivity [24]. In this line, it has been observed that low levels of growth hormone and testosterone have been associated with increased mortality and morbidity in patients with heart failure [25, 26]. Thus, improvement in different cardiovascular indices followed by different aspects of quality of life and psychological status after testosterone therapy can be expectable. In the present study and among study parameters, 6MWD was significantly increased following testosterone prescription. Furthermore, those patients receiving testosterone experienced considerable improvement in quality of life that was not indicated in placebo group. In a similar study by Stout and colleagues, comparable improvements in shuttle walk test, body mass, and hand grip strength from baseline were shown in both testosterone and placebo groups. However, peak oxygen uptake, Beck Depression Inventory, leg strength, and several quality of life components were more improved in former interventional group [27]. Similarly, Iellamo et al. showed that 6MWD, peak oxygen consumption, maximal voluntary contraction, and peak torque increased significantly in testosterone group but remained unchanged in control group [28]. In a recent meta-analysis, a significant increase was shown in exercise capacity following testosterone therapy [29]. Also, it has been suggested that low circulating testosterone independently relates to exercise intolerance that the greater reduction of serum testosterone is accompanied with more severe progression of exercise intolerance [30].

A variety of underlying mechanisms involved in the beneficial effects of testosterone in heart failure condition have been investigated. Some improvement in hemodynamic parameters following injection of testosterone has been demonstrated to be mediated by reduction in peripheral vascular resistance and increased coronary blood flow through vasodilation leading improvement of functional and symptomatic status [31]. Also, some effective role of this therapeutic regimen in preventing the occurrence of serious arrhythmia can be explainable by a direct regulative effect of testosterone on shorten Q-T intervals [32]. Moreover, association between the lack of testosterone and stimulating inflammatory responses has been shown and thus a remarkable improvement of cardiac performance and a significant decrease in the level of serum TNF-alpha following testosterone therapy has been suggested [33]. Along with the pointed mechanisms, a rise in hemoglobin [34], improvement of baroreceptor sensitivity [35], increase of muscle sympathetic nerve activity, and an increase of muscle arteriole vasodilation and function [36] have been also proposed. In addition, because insulin resistance is a major metabolic disturbance occurring in up to 40% of heart failure patients [37], this abnormality has been shown to be associated with low testosterone level leading decreased glucose utilization by skeletal muscle that results in muscle fatigue and wasting [38, 39]. Thus, testosterone therapy can be potentially increase insulin sensitivity and thus improve glucose uptake by muscles and reduced disabilities. It seems that all beneficial cardiovascular, hormonal, and metabolic effects of testosterone injection can explain considerable improvement of quality of life in these patients.

According to the current understanding of testosterone replacement therapy with the goal of achieving testosterone levels that are consistently in the normal range pointed to in ISSAM recommendations [40], we applied low dose of testosterone in our trial. In some other experiments, testosterone enanthate was injected every 10 to 14 days [41]. Thus, we should apply higher dosages of testosterone enanthate with the aim of assessing real effects of this drug on study parameters. However, because of the lack of measuring baseline level of testosterone, nonexistence of monitoring of serum level of the drug, and lack of awareness of the drug effects in heart failure patients with an unusual clinical condition, we preferred to consider lower doses of this drug in our experiment. In addition, despite considering its low dose, useful effects of testosterone therapy on different physical and psychological aspects in heart failure patients were demonstrated.

As a potential limitation, we could not assess baseline testosterone level due to the high cost of these tests in our regions. It seems that the measurement of baseline serum level of testosterone could be useful to assess modification of the study clinical parameters along with monitoring changes in level of testosterone in serum. Furthermore, considering both hypogonadal and eugonadal subjects could result in providing a more comprehensive view of physiological effects of testosterone and other androgen endogenous hormones on cardiovascular parameters in CHF patients.

In conclusion, testosterone deficiency plays the major role in the pathophysiology of congestive heart failure, insights into the beneficial role of testosterone in improvement of function capacity, quality of life, and psychological state in these patients are strengthened. The present study attempted to demonstrate useful effects of testosterone therapy on different physical and psychological aspects in heart failure patients that result in confirming improvement of functional capacity and quality of life in these patients. Improvement in the Tei index consider as an indictor for early improvement in the cardiac function. Since Tei index shows very fine and subtle changes at the beginning of cardiac function so it needs to follow the patients for longer time.

## Figures and Tables

**Table 1:** Comparison of baseline information between intervention and placebo groups.

Characteristics	Testosterone group (*n* = 25)	Placebo group (*n* = 25)	*P* value
Age, year	60.83 ± 8.31	60.24 ± 11.89	0.841
Body mass index, kg/m^2^	26.78 ± 2.91	26.11 ± 3.97	0.505
Diabetes mellitus	7 (28)	6 (24)	0.747
Hypertension	7 (28)	3 (12)	0.289
Hyperlipidemia	11 (44)	6 (24)	0.136
Cigarette smoking	2 (8)	1 (4)	0.998
NYHA classification			0.272
II	20 (80)	13 (52)	
III	5 (20)	10 (40)	
IV	0 (0)	2 (8)	

**Table 2:** Changes in hemodynamic indices, body weight, and function capacity.

Item	At baseline	4 weeks later	8 weeks later	12 weeks later
Systolic blood pressure (mmHg)				
Testosterone group	119.92 ± 16.99	121.60 ± 12.99	122.84 ± 9.49	125.92 ± 15.67
Placebo group	126.12 ± 18.87	122.24 ± 19.17	121.24 ± 18.38	120.74 ± 28.45
*P* value	0.232	0.891	0.700	0.434
Diastolic blood pressure (mmHg)				
Testosterone group	81.17 ± 18.77	75.80 ± 9.70	76.44 ± 7.58	79.00 ± 9.99
Placebo group	79.04 ± 8.94	73.76 ± 8.95	72.68 ± 19.27	76.13 ± 8.26
*P* value	0.613	0.444	0.369	0.286
Heart rate (bpm∗)				
Testosterone group	71.21 ± 8.83	70.48 ± 9.49	69.92 ± 6.84	70.88 ± 8.78
Placebo group	69.52 ± 7.56	71.28 ± 7.12	68.56 ± 8.60	71.52 ± 7.94
*P* value		0.738	0.539	0.792
Body weight (Kg)				
Testosterone group	76.66 ± 10.42	75.88 ± 10.35	75.84 ± 10.26	75.46 ± 9.96
Placebo group	73.12 ± 12.67	74.00 ± 12.47	74.28 ± 11.92	74.52 ± 11.92
*P* value	0.286	0.565	0.622	0.768
6MWD^†^ (m)				
Testosterone group	407.44 ± 100.23	466.58 ± 93.33	476.67 ± 96.59	491.65 ± 112.88
Placebo group	338.25 ± 125.60	394.17 ± 98.37	421.52 ± 99.14	416.09 ± 121.57
*P* value	0.038	0.012	0.044	0.034
Muscle strength (kgf)				
Testosterone group	37.64 ± 9.64	39.60 ± 8.19	41.60 ± 9.57	42.12 ± 9.06
Placebo group	35.17 ± 10.54	36.68 ± 6.87	38.88 ± 7.73	38.65 ± 8.17
*P* value	0.411	0.178	0.278	0.170

*Beats per minute.

^†^Six-minute walk distance.

Data are presented as mean ± SD.

**Table 3:** Improvement in 6MWD following testosterone therapy.

Item	*F*-index∗	*P* value^†^
Testosterone therapy	4.402	0.045
Advanced age	5.135	0.031
Body mass index	6.035	0.020
Diabetes mellitus	1.786	0.192
Hypertension	0.064	0.803
Hyperlipidemia	0.113	0.739
Cigarette smoking	1.203	0.282
NYHA classification	1.592	0.217

Adjusted for baseline variables using the repeated measure ANOVA test.

*F-index: the interpretation of the *F*-index (indicating the level of variability between subjects) is considered significant if it is higher than 0.40.

^†^ *P* values of 0.05 or less for each variable indicated discrepancy in the time trend of the variable between the two groups.

**Table 4:** Changes in cardiovascular parameters, quality of life, and depression score.

Item	Testosterone group		Placebo group		*P* value
	Baseline	Endpoint	Baseline	Endpoint	
Quality of life	128.96 ± 4.91	130.95 ± 2.84	128.00 ± 4.56	127.50 ± 3.44	0.002
Beck score	4.60 ± 4.41	5.00 ± 6.28	4.63 ± 3.14	5.55 ± 5.50	0.152
End diastolic diameter	5.94 ± 1.06	5.86 ± 0.98	5.90 ± 0.82	5.66 ± 0.79	0.440
End systolic diameter	4.92 ± 1.02	4.66 ± 1.06	6.87 ± 1.27	4.49 ± 0.89	0.557
End diastolic volume	141.44 ± 54.06	137.28 ± 48.56	149.16 ± 46.51	130.26 ± 50.72	0.627
End systolic volume	88.72 ± 35.63	87.76 ± 40.80	94.56 ± 43.75	87.04 ± 36.40	0.949
Ejection fraction	34.52 ± 7.39	37.12 ± 8.23	30.76 ± 7.99	35.00 ± 7.91	0.368
Tei index∗	0.54 ± 0.02	0.47 ± 0.01	0.55 ± 0.02	0.59 ± 0.02	0.025

*The pulsed Doppler Tei index is a parameter to evaluate combined systolic and diastolic cardiac performance that is measured by dividing Isovolumic contraction time plus isovolumic relaxation time by ejection time.

Data are presented as mean ± SD.

**Table 5:** Improvement in quality of life following testosterone therapy adjusted for baseline variables using the multivariate linear regression modeling.

Item	Standardized beta∗	*P* value^†^
Testosterone therapy	0.419	0.035
Advanced age	0.319	0.195
Body mass index	0.015	0.936
Diabetes mellitus	0.022	0.912
Hypertension	0.236	0.296
Hyperlipidemia	0.124	0.514
Cigarette smoking	0.062	0.719
NYHA classification	0.070	0.706

*R*-square = 0.436.

*The beta coefficient is used to compare the strength of a predictor within the model to predict the dependant variable or outcome so that the higher beta indicates more power of the variable to predict outcome. Because beta coefficient is potentially affected by the standard error of the variable, the standardized beta adjusted for this error is considered.

^†^A *P* value lees than 0.05 indicates a significant role for predicting outcome by the considered variable.
